# The relationship between estimated GFR based on the CKD-EPI formula and renal inulin clearance in potential kidney donors 

**DOI:** 10.5414/CN108341

**Published:** 2014-10-27

**Authors:** Otto Schück, Vladimir Teplan, Jan Maly, Janka Franekova, Hana Malinska, Milena Stollova, Irena Latova, Jana Urbanova, Jelena Skibova, Ondrej Viklicky

**Affiliations:** 1 Department of Nephrology, Transplant Center,; 2 Division of Professional Outpatient Care,; 3 Specialized Laboratory of Biochemistry, Department of Laboratory Methods,; 4 Department of Metabolism and Diabetes, Center for Experimental Medicine, and; 5 Department of Quality Control and Professional Programs, Division of Institute Management, Institute for Clinical and Experimental Medicine, Prague, Czech Republic

**Keywords:** CKD-EPI, estimated glomerular filtration rate, kidney transplantation, living donor

## Abstract

It is not yet clear whether or not renal function in the living donor can be sufficiently assessed by estimated glomerular filtration rate (GFR) using creatinine-based equations. The present paper investigates the relationship between GFR values determined using renal inulin clearance (Cin) and those estimated using the Chronic Kidney Disease Epidemiology Collaboration (CKD-EPI) formula. Our study was performed in 287 potential kidney donors with a mean age of 48 ± 10 years. Mean Cin was 1.47 ± 0.28 (1.10 – 2.50) mL/s/1.73 m^2^. Total bias when using the CKD-EPI formula was –0.0183 mL/s/1.73 m^2^, precision 0.263 mL/s/1.73 m^2^, and accuracy 90.6% within ± 30% of Cin. The sensitivity of CKD-EPI to estimate a decrease in Cin below 1.33 mL/s/1.73 m^2^ was 50.5%, with an 85% specificity of detecting a value above the cutoff. Receiver-operating curve analysis for the above produced an area under the curve of 0.766 ± 0.0285 (CI 0.712 – 0.813). For donor screening purposes, CKD-EPI should be interpreted with great caution.

## Introduction 

A crucial prerequisite when considering potential kidney donors is an accurate assessment of renal function [1]. The measure used to assess the level of renal function is glomerular filtration rate (GFR), either determined using an exogenous marker (mGFR) or estimated (eGFR), and most often uses formulas based on serum creatinine levels. The GFR sufficient for kidney donation has not yet been conclusively established. Another crucial consideration includes the methods acceptable for determining the cutoff value of GFR while still meeting the above requirements. A GFR generally deemed acceptable for kidney donation is 80 mL/min/1.73 m^2^ = 1.33 mL/s/1.73 m^2^ [2, 3]. While some authors put the cutoff value at 90 mL/min/1.73 m^2^ = 1.50 mL/s/1.73 m^2^ [4], others accept levels as low as 70 – 80 mL/min/1.73 m^2^ = 1.17 – 1.33 mL/s/1.73 m^2^ [5] if carefully considering all other criteria for donation. A GFR of 80 mL/min/1.73 m^2^ (= 1.33 mL/s/1.73 m^2^) is consistent with the value determined by Wesson [6] in healthy adult men and women below the age of 60 years. In these individuals, the GFR as determined by renal inulin clearance (Cin) does not decline below 80 mL/min/1.73 m^2^. A GFR of 80 mL/min/1.73 m^2^ as a minimal value required for kidney donation is also included in the Amsterdam Forum guidelines [7]. 

Another major consideration is the method used to measure or estimate GFR. The question of how closely the results of the various methods for eGFR in individuals with chronic kidney disease (CKD) match the values obtained using exogenous markers is discussed in detail in KDIGO 2013 [8]. In transplantation medicine, the tool most often employed for mGFR is iothalamate clearance (I^125^-labeled or unlabeled). In routine clinical practice, GFR is frequently estimated using creatinine-based equations, most commonly Modification of Diet in Renal Disease (MDRD) [4, 8]. However, this simple equation very often tends to underestimate GFR [9], especially in individuals with a GFR > 60 mL/min/1.73 m^2^ (= 1.0 mL/s/1.73 m^2^), which is an important consideration in the context of kidney donation [4]. A new formula for eGFR developed in recent years and takes into account GFR > 60 mL/min/1.73 m^2^ as well as normal values; this new formula is referred to as Chronic Kidney Disease Epidemiology Collaboration (CKD-EPI) [10]. A comparison of eGFR using CKD-EPI with iothalamate clearance (I^125^-labeled) in living kidney donors exhibited a smaller bias and higher precision and accuracy than the MDRD equation [3]. As a result, some authors recommend use of CKD-EPI in assessing GFR in living kidney donors [4]; however, other authors [3] point out that the results obtained with all creatinine-based equations for eGFR (including CKD-EPI) should be interpreted with great caution given the higher specificity of the CKD-EPI formula as compared with MDRD [3]. 

It has not yet been clearly established whether the CKD-EPI formula is sufficient for measuring GFR in living donors. Our study was therefore designed to determine, in potential kidney donors, the relationship between the GFR established using renal Cin (under conditions of stabilized plasma concentrations) and the eGFR calculated using the CKD-EPI formula. Special attention was given to whether or not the eGFR calculated using the CKD-EPI formula could help to evaluate whether the GFR in the examined individual was below the acceptable cutoff value for kidney donation set at 80 mL/min/1.73 m^2^ = 1.33 mL/s/1.73 m^2^. 

## Methods 

Renal function was assessed in 287 healthy adults. Demographics and baseline characteristics are shown in Table 1. Clinical examination and routine laboratory tests [1, 11] had to be within normal limits. Key exclusion criteria included relevant concomitant disease, including psychiatric disorders or drug abuse. Subjects were informed about the aims and risks of kidney donation. 

The study was performed in accordance with the Declaration of Helsinki. 

### Renal function assessment 

In addition to the determination of serum creatinine (SCr) levels, chemical and microscopic urinalysis, all patients had their renal Cin determined during the morning hours in a quiet, separate room. Inulin was administered in the form of polyfructosan (Inutest, Fresenius Kabi, Graz, Austria). A loading dose of inulin was injected into a peripheral vein (50 mg/kg). Immediately after the injection, a cannula was connected to a microinfusion pump operating at a rate 0.20 mL/minute, which maintained the plasma levels of inulin within the range 200 – 300 mg/L. Half an hour before the examination, 10 mL/kg of water was provided. Diuresis amounted to a minimum 3 mL/minute. Any adverse reactions of administering inulin were not recorded. 

The collection period lasted 60 – 90 minutes. This examination is described in detail in our earlier paper [12]. 

### Analytical methods 

Serum and urinary inulin levels were determined by a spectrophotometric technique developed by White and Samson [13]. The coefficient of variation (CV) of this method (validated at 3 different plasma and urinary levels) did not exceed 2%. 

SCr levels were determined using an enzymatic method (Abbot Architect Creatinine, catalogue 8L24-31, Abbot Laboratories Inc., Abbot Park, IL, USA) standardized by NISTSRM 957. The CV of this method for lower creatinine levels (117 µmol/L) was 0.32%, and it was 0.3% for higher levels (268.5 µmol/L). The CKD-EPI value was calculated using the formula developed by Levey et al. [10]. 

### Statistical analysis 

The results were given as mean and standard deviation (SD). The values obtained were used to calculate total bias, precision, and accuracy. Agreement between the tested and referenced methods was analyzed using the method developed by Bland and Altman [14]. The relationship between the studied measures was calculated by regression analysis. Receiver operating characteristic (ROC) plots and analyses were performed using Med Calc software, version 12.2.1 (Mariakerke, Belgium). Statistical analysis was performed using BMDP Statistical Software, Release 8.1 (Statistical Solutions Ltd., Cork, Ireland). 

## Results 

The mean value of Scr levels in our group was 75.4 ± 10.8 (42 – 103) µmol/L, with a mean Cin of 1.47 ± 0.28 (1.10 – 2.50) mL/s/1.73 m^2^. Mean calculated CKD-EPI-derived GFR was 1.48 ± 0.25 (0.92 – 2.25) mL/s/1.73 m^2^. Mean total bias was –0.0183 mL/s/1.73 m^2^, and precision was 0.263 mL/s/1.73 m^2^. In the overall population, accuracy values within ± 15, ± 30, and ± 50% difference from Cin for CKD-EPI were 55.9, 90.6, and 99.3%, respectively. 

**Figure 1.**
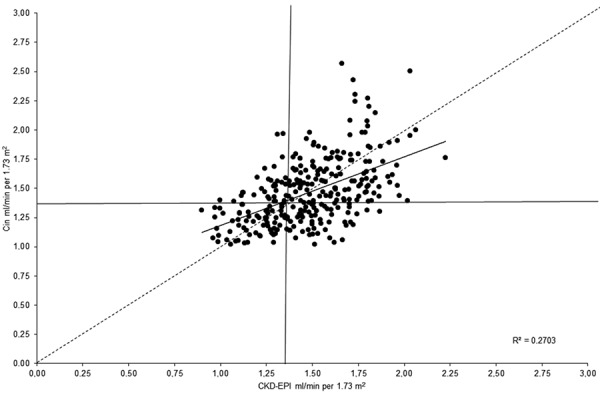
The relationship between the values obtained by the CKD-EPI formula and renal inulin clearance.

The relationship between the values obtained using the CKD-EPI formula and Cin is depicted in Figure 1, clearly showing a correlation between both measures (r = 0.520; p < 0.001). The entire area of the graph is divided into 4 fields by a vertical line running at the level of CKD-EPI = 1.33 mL/s/1.73 m^2^ and a horizontal line for the same Cin value. The above cutoff value was used to assess the sensitivity and specificity of CKD-EPI. 

**Figure 2.**
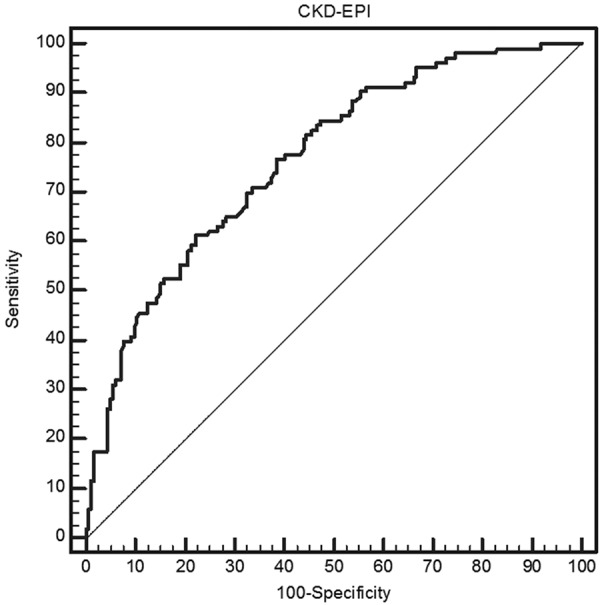
Receiver operating characteristic (ROC) curve for CKD-EPI. Cutoff  value for Cin = 1.33 mL/s/1.73 m^2^.

The sensitivity and specificity of CKD-EPI are 50.5% and 85.0%, respectively. ROC analysis for a Cin cutoff value of 1.33 mL/s/1.73 m^2^ showed an area under the curve (AUC) of 0.766 ± 0.0285 (CI 0.712 – 0.813) (Figure 2). 

**Figure 3.**
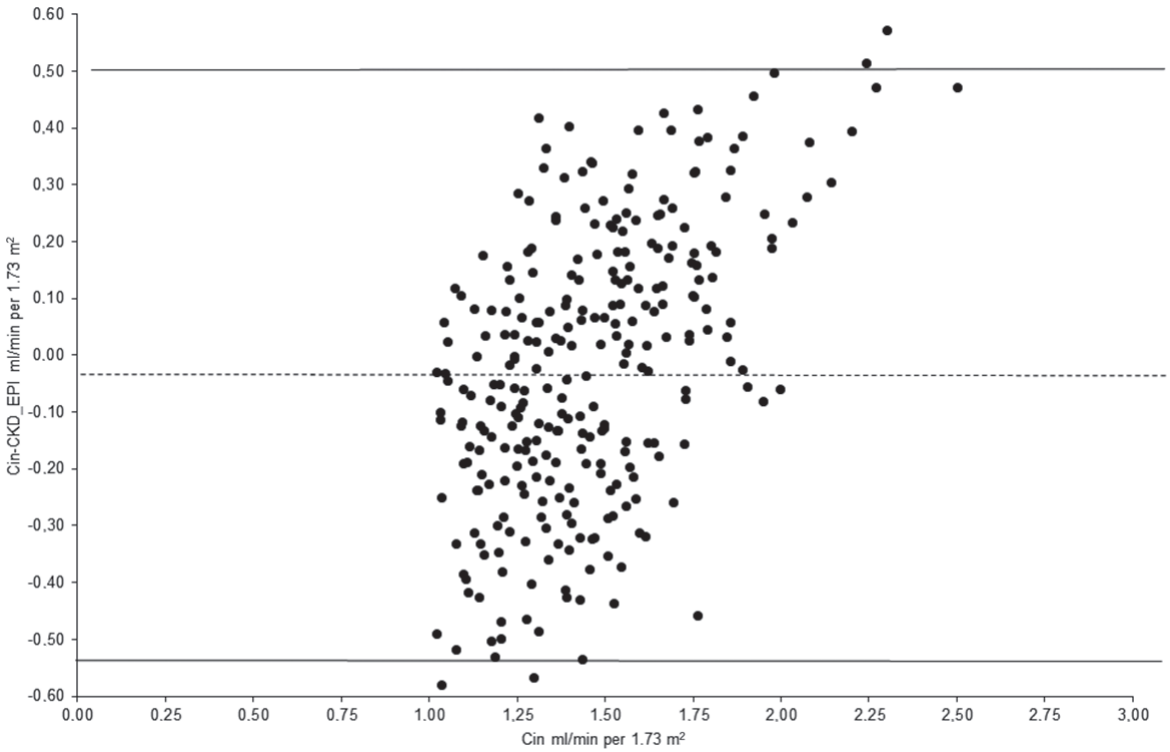
Bland and Altman plots comparing Cin – CKD-EPI and Cin.

The 2SD of the difference between Cin and CKD-EPI measures was 0.52 mL/s/1.73 m^2^ (Figure 3) when assessing for agreement. 

## Discussion 

The results obtained suggest a significant correlation between the values of Cin and those estimated using the CKD-EPI formula. This significant correlation could be documented despite the absence of very low mGFR in our group of examined individuals; their presence would have no doubt raised the significance of the correlation between Cin and CKD-EPI-derived values. The lowest Cin values in our group were never below 1.0 mL/s/1.73 m^2^. Despite the significant correlation between the two measures, analysis of agreement produced a relatively high 2SD (0.52 mL/s/1.73 m^2^) suggesting that eGFR using the CKD-EPI formula cannot be used as an alternative to accurate GFR determination in potential kidney donors. 

This assumption is in keeping with the findings reported by Tent et al. [3] investigating the relationship between eGFR using Scr-based formulas (including CKD-EPI) and iothalamate clearance. The same conclusion was made by Murata et al. [15]. By contrast, Lujan et al. [4], also studying the relationship between CKD-EPI-derived values and iothalamate clearance, believe that the CKD-EPI formula can be employed to assess GFR in living kidney donors. 

Our results obtained by analyzing the relationship between CKD-EPI-derived values and Cin under conditions of stabilized plasma levels support the following conclusions. The sensitivity of CKD-EPI to detect a decrease in GFR below 1.33 mL/s/1.73 m^2^ (80 mL/min/1.73 m^2^) is very low (50.5%). The implication is that, at CKD-EPI-derived GFR levels < 1.33 mL/s/1.73 m^2^, it is impossible to determine whether or not GFR is indeed reduced. The probability of GFR exceeding this cutoff value is virtually identical to the probability of being lower. By contrast, the specificity of CKD-EPI is clearly superior to its sensitivity. At CKD-EPI-derived GFR levels > 1.33 mL/s/1.73 m^2^, there is an 85% probability that the GFR calculated using Cin exceeds the above value; however, a GFR reduced below the arbitrary cutoff value in 15% of cases cannot be ruled out. These findings support the assumption that the CKD-EPI formula can only be useful in potential kidney donors at values higher than 1.33 mL/s/1.73 m^2^. Therefore, one should ask whether or not the specificity of this method is sufficient for determining GFR in living donors. The answer to this question is not an easy one. Obviously, the risk of losing one kidney for the living kidney donor should be kept at a minimum [2]. However, this contrasts with the need to increase the number of living kidney donors combined with expanding the criteria for kidney donation. Our results are in keeping with the assumption that for donor screening purposes the CKD-EPI equation should be interpreted with great caution. A novel method recommended for determining GFR in living donors, in addition to renal iothalamate clearance, is (99m) Tc-DTPA renal dynamic imaging [5]. In this study, Cin has been used instead of iothalamate clearance because small tubular secretion of iothalamate cannot be excluded [16, 17]. The relationship between the creatinine clearance based formula derived for Japanese people and Cin are in accordance with the results obtained for the Caucasian population. The Japanese GFR estimation equation did not accurately estimate mGFR in Japanese living donors [18]. Still, it is renal Cin which remains the gold standard in measuring glomerular filtration rate [8]. 

## Funding source 

The study was supported by a grant awarded by the Internal Grant Agency of the Ministry of Health of the Czech Republic (NT 13139-3/2012) and by MH CZ - DRO (“Institute for Clinical and Experimental Medicine – IKEM, IN 00023001”). 

## Conflict of interest 

The authors have declared no conflicts of interest. 

**Table 1.** Subject demographics and baseline characteristics.

Age (years)	Mean (SD)	48.6 (10.3)
	Range	21.4 – 70.1
Gender (n %)	Male	105 (36.6)
	Female	182 (63.4)
Race	Caucasian	
BMI (kg/m^2^)	Mean (SD)	26.2 (1.8)
	Range	18.3 – 29.4
Scr (µmol/L)	Mean (SD)	75.4 (10.8)
	Range	42.0 – 103.1

SD = standard deviation; BMI = body mass index; SCr = serum creatinine.

## References

[1] Guideline on the management and evaluation of the kidney donor and recipient. Nephrol Dial Transplant. 2013; 28: ii1–ii71. 24026881 10.1093/ndt/gft218

[2] Hourmant M Lerat L Karam G Donation from old living donors: how safe is it? Nephrol Dial Transplant. 2013; 28: 2010–2014. 23543591 10.1093/ndt/gft069

[3] Tent H Rook M Stevens LA van Son WJ van Pelt LJ Hofker HS Ploeg RJ van der Heide JJ Navis G Renal function equations before and after living kidney donation: a within-individual comparison of performance at different levels of renal function. Clin J Am Soc Nephrol. 2010; 5: 1960–1968. 20616162 10.2215/CJN.08761209 PMC3001772

[4] Lujan PR Chiurchiu C Douthat W de Arteaga J de la Fuente J Capra RH Massari PU CKD-EPI instead of MDRD for candidates to kidney donation. Transplantation. 2012; 94: 637–641. 22918217 10.1097/TP.0b013e3182603260

[5] Zhao X Shao Y Wang Y Tian J Sun B Ru Y Zhang A Hao J New normal values not related to age and sex, of glomerular filtration rate by (99m)Tc-DTPA renal dynamic imaging, for the evaluation of living kidney graft donors. Hell J Nucl Med. 2012; 15: 210–214. 23106053 10.1967/s002449910057

[6] Wesson L Physiology of the human kidney. New York, NY: Grune and Stratton; 1969.

[7] Delmonico F A Report of the Amsterdam Forum On the Care of the Live Kidney Donor: Data and Medical Guidelines. Transplantation. 2005; 79: S53–S66. 15785361

[8] KDIGO 2012 Clinical Practice Guideline for the Evaluation and Management of Chronic Kidney Disease. Kidney inter. Suppl.. 2013; 3: 1–150. 10.1038/ki.2013.243 23989362

[9] Botev R Mallié JP Couchoud C Schück O Fauvel JP Wetzels JF Lee N De Santo NG Cirillo M Estimating glomerular filtration rate: Cockcroft-Gault and Modification of Diet in Renal Disease formulas compared to renal inulin clearance. Clin J Am Soc Nephrol. 2009; 4: 899–906. 19406960 10.2215/CJN.05371008 PMC2676189

[10] Levey AS Stevens LA Schmid CH Zhang YL Castro AF Feldman HI Kusek JW Eggers P Van Lente F Greene T Coresh J A new equation to estimate glomerular filtration rate. Ann Intern Med. 2009; 150: 604–612. 19414839 10.7326/0003-4819-150-9-200905050-00006 PMC2763564

[11] Trevitt R Living kidney donors: the need to minimise long term risk. J Ren Care. 2011; 37: 134–147. 21810195 10.1111/j.1755-6686.2011.00225.x

[12] Schück O Examination of Kidney Function. Boston: Martinus Nijhoff; 1984.

[13] White RP Samson FE Determination of inulin in plasma and urine by use of anthrone. J Lab Clin Med. 1954; 43: 475–478. 13143338

[14] Bland JM Altman DG Statistical methods for assessing agreement between two methods of clinical measurement. Lancet. 1986; 1: 307–310. 2868172

[15] Murata K Baumann NA Saenger AK Larson TS Rule AD Lieske JC Relative performance of the MDRD and CKD-EPI equations for estimating glomerular filtration rate among patients with varied clinical presentations. Clin J Am Soc Nephrol. 2011; 6: 1963–1972. 21737852 10.2215/CJN.02300311 PMC3156428

[16] Skov PE Glomerular filtration rate in patients with severe and very severe renal insufficiensy: Determined by simultaneous inulin, creatine and 125 iothalamate clearance. Acta Med Scand. 1970; 187: 419–428. 5526960 10.1111/j.0954-6820.1970.tb02965.x

[17] Botev R Mallié JP Wetzels JF Couchoud C Schück O The clinician and estimation of glomerular filtration rate by creatinine-based formulas: current limitations and quo vadis. Clin J Am Soc Nephrol. 2011; 6: 937–950. 21454722 10.2215/CJN.09241010

[18] Kakuta Y Okumi M Ichimaru N Abe T Nonomura N Okuyama A Kojima Y Isaka Y Takahara S Imai E Horio M Utility of the Japanese GFR estimation equation for evaluating potential donor kidney function. Clin Exp Nephrol. 2010; 14: 63–67. 19806425 10.1007/s10157-009-0224-0

